# A Review of Systemic Minocycline Side Effects and Topical Minocycline as a Safer Alternative for Treating Acne and Rosacea

**DOI:** 10.3390/antibiotics10070757

**Published:** 2021-06-22

**Authors:** Ana M. Martins, Joana M. Marto, Jodi L. Johnson, Emmy M. Graber

**Affiliations:** 1 Research Institute for Medicine (iMed.ULisboa), Faculty of Pharmacy, Universidade de Lisboa, 1649-003 Lisbon, Portugal; amartins@farm-id.pt (A.M.M.); jmmarto@ff.ulisboa.pt (J.M.M.); 2 Departments of Pathology and Dermatology, Feinberg School of Medicine, Northwestern University, Chicago, IL 60611, USA; jodi-johnson@northwestern.edu; 3 The Dermatology Institute, Boston, MA 02116, USA; 4 Northeastern University, Boston, MA 02115, USA

**Keywords:** minocycline, tetracycline, acne vulgaris, rosacea

## Abstract

Resistance of *Cutibacterium acnes* to topical antibiotics historically used to treat acne (topical erythromycin and clindamycin and, more recently, topical azithromycin and clarithromycin) has been steadily increasing and new topical antibiotics are needed. Minocycline is a semisynthetic tetracycline-derived antibiotic currently used systemically to treat a wide range of infections caused by Gram-negative and Gram-positive bacteria. In addition to its antibiotic activity, minocycline possesses anti-inflammatory properties, such as the downregulation of proinflammatory cytokine production, suppression of neutrophil chemotaxis, activation of superoxide dismutase, and inhibition of phagocytosis, among others. These characteristics make minocycline a valuable agent for treatment of dermatological diseases such as acne vulgaris and papulopustular rosacea. However, more frequent or serious adverse effects have been observed upon the systemic administration of minocycline than with other tetracyclines. Examples of serious adverse effects include hypersensitivity syndrome reaction, drug-induced lupus, idiopathic intracranial hypertension, and other autoimmune syndromes that may cause death. Here, we review adverse effects and drug–drug interactions observed with oral administration of minocycline and contrast this with topical minocycline formulations recently approved or under development for effectively treating dermatological disorders with fewer adverse effects and less drug interaction.

## 1. Introduction

**Figure 1:**
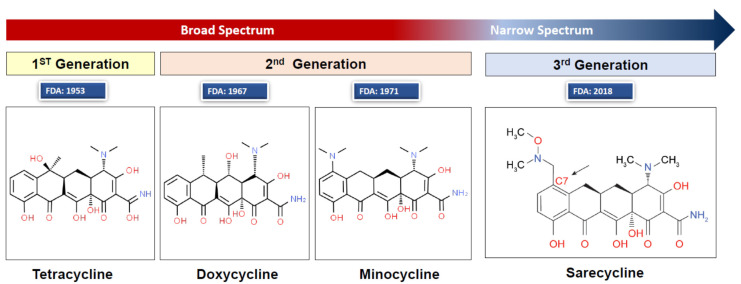
Chemical structures of tetracycline, doxycycline, minocycline, and sarecycline. Structure image source: http://www.chemspider.com/Chemical-Structure.28540486.html (accessed on 10 January 2020). Image created and provided with permission by Almirall LCC, Exton, PA, USA.

The first tetracyclines were discovered in the 1940s as natural products of *Streptomyces* strains [1]. Tetracycline is the first-generation drug in the tetracycline family and was approved by the Food and Drug Administration (FDA) in 1953. Minocycline and doxycycline are chemically modified, second-generation, broad-spectrum tetracyclines, which, compared to the first-generation, have enhanced pharmacodynamic properties, improved antimicrobial activities, and favorable clinical experience [2]. Sarecycline is a third-generation tetracycline with a narrower spectrum of antimicrobial activity [3,4]. All the semisynthetic tetracycline-based drugs consist of a linear fused tetracyclic nucleus (4 rings, A–D) to which several functional groups are attached [5]. Minocycline has modifications to carbons 7–9 on ring D, which gives it specific antibacterial properties [6]. The chemical formula of minocycline is [4S(4α,4aα,5aα,12aα)]-4,7-bis(dimethylamino)-1,4,4a,5,5a,6,11,12a-octahydro-3,10,12,12a-tetrahydroxy-1,11-dioxo-2-naphthacenecarboxamide mono hydrochloride (C_23_H_27_N_3_O_7_.HCl, MW = 493.94) (Figure 1).

The antibacterial activity of minocycline was first described in 1966 [7] and a form of minocycline (Solodyn minocycline hydrochloride extended-release tablets) was approved by the FDA for treatment of inflammatory lesions of non-nodular moderate-to-severe acne vulgaris in patients 12 years and older in 2006 [8]. The second-generation tetracyclines (minocycline and doxycycline) have a broad spectrum of activity, effectively targeting a wide range of Gram-positive and Gram-negative bacteria, atypical organisms such as chlamydiae, mycoplasmas, rickettsiae, and protozoan parasites [5]. Many strains of Gram-positive bacteria are resistant to tetracyclines; thus, culture and susceptibility tests are recommended prior to use. 

As a broad-spectrum antibiotic, minocycline is used to treat many bacterial infections, such as urinary tract infections, respiratory infections, skin infections (such as moderate-to-severe acne), chlamydia, tick fever, and others. It is also used for gonorrhea, syphilis, and other infections as a second-line drug in patients with allergy to penicillin [9].

Minocycline is primarily bacteriostatic, with a mechanism of action similar to other tetracycline antibiotics, i.e., inhibition of bacterial protein biosynthesis via binding to the 30S ribosomal subunit and inhibiting the ligation of the aminoacyl-tRNA [5,6]. In addition to its antibiotic action, minocycline has potent anti-inflammatory properties [6], which become important when minocycline is chosen for treatment of skin disorders as discussed below.

## 2. Systemic Minocycline for the Treatment of Dermatological Diseases

Minocycline is effective for treatment of various inflammatory skin disorders, including acne, rosacea, bullous dermatoses, and neutrophilic dermatoses [10]. Acne and rosacea are amongst the most common conditions treated by dermatologists and the use of minocycline in treating these conditions will be highlighted here. 

### 2.1. Rosacea and Acne Vulgaris 

Rosacea and acne vulgaris present therapeutic challenges due to their chronicity, potential for disfigurement and psychosocial impact. Although pathophysiologically distinct, both conditions have major inflammatory components. Consequently, systemic antimicrobial agents are routinely prescribed for months at a time rather than for days or weeks as is typical with infections. Emergence of resistant bacterial strains, adverse events and compliance issues associated with chronic systemic broad-spectrum antibiotic use have led to new treatment approaches, such as the use of: subantimicrobial doses [11], narrow-spectrum oral antibiotics (i.e., sarecycline), and new topical formulations of minocycline.

Rosacea is a chronic inflammatory skin disease occurring mostly in adults and affecting about 415 million people worldwide [12]. Rosacea is classified into 4 subtypes: erythematotelangiectatic, papulopustular, phymatous, and ocular. Some patients exhibit more than one subtype concomitantly. Clinical findings of rosacea may include flushing (transient erythema), permanent fixed erythema, telangiectasias, papules, and/or pustules. Skin findings are most often pronounced on the central face, particularly in areas with a convex surface such as the nose, medial cheeks, and chin [13,14]. Rosacea patients have a compromised skin barrier and therefore often experience burning, stinging, itching, and/or sensitivity to facial products and cosmetics. Rosacea can negatively impact a patients’ quality of life causing anxiety, depression and low self-esteem, and self-confidence [15]. 

The cause of rosacea is unknown but it is suspected to be multifactorial with genetic factors and environmental components playing a role. A damaged skin barrier may also play a role in initiating or augmenting rosacea [16]. In fact, those with papulopustular rosacea (PPR) have skin barrier damage similar to those with atopic dermatitis [16]. An intact skin barrier maintains homeostasis by protecting the body from microbes and preserving a waterproof shell [16]. Rosacea skin has an increased pH, greater transepidermal water loss, and decreased skin hydration levels compared to unaffected skin [17,18]. 

Those with rosacea note that their skin symptoms and appearance vary day-to-day and even oscillate throughout the day. Triggers of rosacea may include: temperature extremes (either heat or cold), exercise, sunlight, spicy foods, alcohol, menopause, stress, and use of steroid creams on the face. Triggers dysregulate the innate and adaptive immune systems, causing release of several mediators from skin immunocyte and nonimmunocyte cells, and can also interact with the cutaneous nervous system, culminating in appearance of the typical rosacea lesions [13]. 

Acne vulgaris is a multifactorial disease affecting the skin pilosebaceous unit and may lead to scarring [19]. Acne most commonly occurs during the teenage years, although adults may be afflicted as well, and has a lifetime prevalence of about 85%. Even though acne lesions are typically classified as either noninflammatory (comedones) or inflammatory (papules and pustules), inflammation is present in all types of acne lesions and even in areas of dyspigmentation where acne lesions once existed (i.e., post inflammatory hyperpigmentation). Although the presence of inflammation seems to be pervasive in acne, several factors contribute to acne pathogenesis. These include: microbial colonization with *Cutibacterium acnes* (formerly *Propionibacterium acnes*) (*C. acnes*)*,* alteration of follicular hyperkeratinization, altered sebum production under androgen control, complex inflammatory mechanisms involving innate and adaptive immune systems, and neuroendocrine regulatory mechanisms [20,21]. Like rosacea, acne negatively impacts patient quality of life leading to low self-esteem, depression, and social isolation [22].

### 2.2. Antibiotic Resistance to Topical Macrolide Antibiotics Historically Used to Treat Acne

Topical clindamycin, erythromycin, azithromycin, and clarithromycin have been used globally to treat acne [23]. However, evidence indicates that antibiotic resistance of *C. acnes* to clindamycin and erythromycin has remained prevalent or increased over the last four decades, particularly in regions like Spain, India, and Egypt. While resistance to azithromycin and clarithromycin has only begun to be sampled in the last decade, the prevalence of *C. acnes* resistance for these two antibiotics is as high as 100% in India and China, with 82% resistance to azithromycin, and 68% resistance to clarithromycin in Mexico [23]. Antibiotic resistance can lead to treatment failure, disturbance of microbiota, local and systemic increase in opportunistic pathogens, and transfer of resistant strains within medical facilities and to the general public. Increased care must be taken when prescribing these antibiotics and development of new topical antibiotics is warranted. 

### 2.3. Treatment of Acne and Rosacea with the Tetracycline Class Antibiotics

Antibiotics, particularly of the tetracycline family, are considered first-line treatments for moderate-to-severe acne [24] and rosacea [25]. Doxycycline and minocycline are the most common oral tetracycline antibiotics prescribed by dermatologists [26]. 

The efficacy of these antibiotics results from a combination of their antimicrobial effect (over *C. acnes* in the case of acne), and their anti-inflammatory properties [24,27]. Tetracyclines downregulate *C. acnes* lipases, preventing the release of follicular fatty acids. This suppresses neutrophil chemotaxis to the follicular site and inhibits phospholipase A2-dependent activation of inflammatory pathways (less inflammation-related tissue damage), decreases metalloproteinases (protects the extracellular matrix from breaking down), and decreases nitric oxide activity (reduces oxidative damage) [27,28]. Tetracyclines, particularly minocycline, also downregulate production of proinflammatory cytokines (e.g., TNF-α, IL-1β, IL-6). These anti-inflammatory and antioxidant properties, as well as the inhibition of collagenase, are important for the management of skin diseases [29]. When compared to tetracycline and doxycycline in vivo, minocycline is more effective against *C. acnes*, which may be due to minocycline’s high lipophilicity, allowing it to achieve higher sebaceous follicular concentrations compared to other tetracyclines [30]. 

### 2.4. Minocycline Dosage Forms Available in the United States

In the USA, systemic minocycline is available as oral tablets, capsules, and subgingival microspheres and powder for intravenous (IV) solution (Table 1). Oral minocycline formulations are used to treat acne vulgaris and rosacea lesions, while the subgingival microspheres are used as an adjunct in the treatment of periodontitis. Oral and IV formulations are used to treat infections by microorganisms sensitive to minocycline [31].

### 2.5. Pharmacokinetics of Systemic Minocycline

Oral minocycline is nearly completely absorbed in the upper part of the small intestine and is widely distributed to body fluids, bile, and tissues, reaching very high concentrations in the gallbladder and liver [33,34]. Minocycline can reach therapeutically relevant concentrations in the cerebrospinal fluid, since it crosses the blood–brain barrier better than other tetracyclines due to its lipophilicity [33,35]. The time to reach highest blood plasma concentrations depends on the dosage form: it ranges from 1 to 3 h for the oral tablets, 1 to 4 h for the pellet-filled capsules, and 3.5 to 4 h for the extended-release tablets [34]. The biological half-life of minocycline ranges from 11–26 h in healthy individuals [9,33], up to 30 h [9] in those with renal dysfunction, and even longer for patients with hepatic disease [33]. About 50% of the drug is metabolized to inactive metabolites in the liver and is mainly eliminated in the feces (20–34%) [36], but is also eliminated via the kidneys (5–15%) [33,34].

### 2.6. Efficacy of Systemic Minocycline for Treatment of Acne and Rosacea

Two 12-week, randomized placebo-controlled trials utilizing extended-release minocycline demonstrated efficacy in treating acne. In total, 615 subjects were treated with extended-release minocycline 1mg/kg daily and compared with 309 subjects treated with a placebo. In both studies, patients treated with minocycline had a significant mean percent reduction in inflammatory lesion count (43.1% and 45.8%) compared to the placebo (31.7% and 30.8%). When graded utilizing an Evaluator’s Global Severity Assessment scale, 16.6% of patient in the minocycline group had treatment success compared to 8.7% of patients in the placebo group [37]. In another trial of 332 inflammatory acne patients, ages 12 years and older, researchers found that, at 3 months, those prescribed 100 mg minocycline daily experienced a 66.55% reduction in inflammatory lesions as compared to a 49.84% reduction in those treated with 30 mg zinc daily (*p* < 0.01) [38]. With regard to treating rosacea, a 16-week study comparing doxycycline (40 mg once daily) with minocycline (100 mg once daily) found that both treatments resulted in similar reductions in rosacea lesion counts.

## 3. Adverse Effects Caused by Systemic Minocycline and Contraindications

### 3.1. Adverse Effects of Oral Minocycline

The most common concern of long-term systemic minocycline use for acne is the development of resistant microorganisms. Minocycline has a lower rate of *C. acnes* resistance compared to tetracycline and doxycycline [27], and *C. acnes* strains exhibited similar spontaneous mutational frequencies when comparing sarecycline, vancomycin, and minocycline [4]. Gastrointestinal dysbiosis, or disruption of the normal gut microbiome homeostasis, is also a concern of long-term use of broad-spectrum oral antibiotics. The gut microbiome is made up of both commensal and pathogenic bacteria that can be disrupted and unbalanced by use of broad-spectrum antibiotics [24]. Even using broad-spectrum oral antibiotics for as little as seven days can result in gut dysbiosis for up to two years [39]. Symptoms related to disruption of the gut microbiome include diarrhea, epigastric discomfort, nausea, and vomiting [40]. Gut dysbiosis may include depletion of gut bacterial diversity and may lead to: compromised immune system homeostasis, increased susceptibility to infections, accumulation of antibiotic resistance, and a dysregulated metabolism [41]. Additionally, due to the broad spectrum of activity of minocycline, yeast overgrowth and resultant yeast infections such as oral and vulvovaginal candidiasis may develop [42]. 

Oral minocycline has more side effects and more severe side effects than other tetracyclines, estimated to occur in 13.6% of all treated patients [43,44,45,46]. Women are generally more prone to minocycline side effects than men [47,48], with ~58% affected females versus 34% males [49]. People 18–44 years old are most affected, corresponding to about 41% of the reported cases, followed by 12–17 years and 45–64 years age groups, both with 15% of cases [49]. A systematic review by Garner et al. [43] found that minocycline led to early onset dose-related toxicity reactions concerning single-organ dysfunctions. The onset of other multiorgan adverse effects is variable, as discussed in the following sections. The adverse effects of 150 mg vs. 200 mg minocycline were largely equivalent [50] and Garner et al. [43] found no evidence that adverse effects are dose-dependent.

Adverse effects from systemic minocycline can afflict the nervous, gastrointestinal, musculoskeletal, respiratory, cutaneous, and genitourinary systems, among others (Table 2) [9]. Since minocycline is metabolized mainly in the liver, unlike other tetracyclines, severe side effects may be due to alterations of cytochrome P450 [51]. However, it is important to emphasize that minocycline has been used extensively for more than four decades and serious side effects are rare [52]. 

#### 3.1.1. Nervous System-Related 

Unlike the other tetracyclines, oral minocycline can cross the blood–brain barrier and therefore may induce nervous system-related side effects. Vestibular side effects such as dizziness, tinnitus, and vertigo have been described for minocycline (between 1% to 10% of patients) but not for doxycycline [54]. The most common adverse effect of oral minocycline is headache (23% of patients) [9]. Uncommonly (less than 0.01% to 0.1% of patients), a minocycline-induced headache might be an initial symptom of a rare but serious syndrome called benign intracranial hypertension, also called pseudotumor cerebri (PTC) or idiopathic intracranial hypertension (IIH) [21]. IIH leads to high intracranial pressure and papilledema caused by the buildup or poor reabsorption of cerebrospinal fluid. A headache associated with IIH is worsened when the patient bends over, coughs, bears down, and sneezes. The symptoms closely mimic those of a brain tumor: headache, vomiting, nausea, blurred vision, and pulsating sounds within the head [55,56]. If suspected, immediate ophthalmologic consultation should be sought to evaluate for papilledema, which can aid diagnosis [57].

The prognosis of IIH related to minocycline is quite variable. Once diagnosed, a lumbar puncture often resolves IIH by alleviating the pressure. While some authors report a benign condition, which disappears spontaneously upon interruption of the offending agent, others report more severe cases involving permanent vision loss [58]. Fraser et al. [59] reported a rapid onset case of IIH in a 12-year-old girl being treated with oral minocycline for acne vulgaris, which led to permanent vision loss despite minocycline interruption and interventions such as a lumbar puncture and surgery with optic nerve sheath fenestration. It must be mentioned, however, that the patient had a medical history of macrocephaly and a family history of hydrocephaly. A similar case was reported by Donnet et al. [56] of a 16-year old girl undergoing oral minocycline treatment for acne. She was diagnosed with IIH [57], which ameliorated once minocycline was ceased, although abnormalities in the visual field remained. Several other case reports can be found in a recent literature review on medication-induced IIH. In summary, IIH is a serious adverse effect of systemic minocycline. 

Isotretinoin has also been reported to cause IIH and therefore it is not recommended that oral minocycline, or any of the oral tetracyclines, be taken concomitantly with isotretinoin. Although it is not known if the risk of IIH is additive if taking both isotretinoin and oral tetracyclines, IIH has been reported in patients taking both agents simultaneously [60]. 

Another nervous system-related adverse effect described with minocycline use is transient depersonalization symptoms (TDS). During a TDS episode, patients feel detached from themselves, with emotional numbness, subjective recall, and derealization [61]. Minocycline-induced depersonalization has been hypothesized to be caused by hypersensitivity of the serotonergic system, drug-related metabolic encephalopathy, drug-induced temporal disintegration, and panic disorder-related etiology [62]. Cohen [62] reported the case of a young female patient who experienced TDS after initiating minocycline treatment, which ceased when the therapy was stopped and resumed when minocycline therapy was reinitiated. A similar case was described by Shamout et al. [63] of a 37-year-old female patient who was prescribed minocycline to treat periorificial dermatitis. The patient did not have any previous history of psychiatric disorders but started experiencing TDS 2 days after starting the minocycline treatment. Just like in the previous case, the symptoms subsided 2 to 3 days after stopping minocycline. 

#### 3.1.2. Skin-Related

Although phototoxicity [64] and photo-onycholysis are common with tetracycline and doxycycline, incidence with minocycline and sarecycline use is low [65,66]. Hyperpigmentation is a well-document and cosmetically unpleasant side effect of oral minocycline use. It may appear as a blue-grey or brown hyperpigmentation and occurs in 3 to 15% of patients. Although reported with short term minocycline use [67], higher incidence occurs with higher doses of minocycline for long periods of time [21,68]. Besides affecting the skin, the hyperpigmentation may also affect bones, nails, teeth and oral mucosa, subcutaneous fat, conjunctiva and sclera, the ear tympanic membrane, and even internal organs [68,69].

There are different types of minocycline-induced skin pigmentation [68,70,71]. The most common, type I, consists of blue-black/grey pigment on the face in areas of scarring or inflammation associated with acne. It is caused by deposition of pigmented granules made of iron chelates of minocycline or melanin present extracellularly or intracellularly in macrophages within the dermis. Type II consists of blue-grey pigmentation of normal skin on the shins and forearms, possibly related to the deposition of pigmented metabolites of minocycline. In type III, muddy-brown skin discoloration occurs in sun-exposed areas, such as the face, possibly due to increased levels of melanin in keratinocytes and epidermal and dermal macrophages. Type IV occurs in areas with scars. Minocycline-induced scleral and, less frequently, conjunctival pigmentation can also occur, especially in older patients who underwent long-term therapy [72,73]. Yokoi et al. [74] reported a case of minocycline-induced hyperpigmentation mimicking angiosarcoma, a life-threatening skin malignancy. Melanoglossia (black tongue) has also been described as a side effect of minocycline [75,76], with symptoms subsiding after discontinuing the treatment. Hyperpigmentation of any type (aside from melanoglossia) is often permanent or takes years to fade. Topical agents are ineffective and lasers and light devices offer poor results for removing minocycline-induced hyperpigmentation.

#### 3.1.3. Dentition-Related

As mentioned above, minocycline use during pregnancy or in children under 9 years old can result in blackened teeth in the infant as teeth develop or in the child as permanent teeth are developing [9,33,77]. Teeth staining can also occur in previously normally colored adult teeth with a reported incidence of 3–6% of patients taking long-term minocycline at >100 mg daily [78]. Onset of discoloration can occur from 1 month to years after taking minocycline. To potentially prevent this side effect, the minocycline dose can be reduced to below 100 mg per day or minocycline can be taken together with Vitamin C or another antioxidant [78]. There are few ways to treat the tooth discoloration once it has occurred, but bleaching, veneers, or crowns may be options.

#### 3.1.4. Autoimmune-Related

Minocycline or one of its metabolites may act as a “superantigen” leading to overactivation of the immune system, namely of lymphocytes, and release of proinflammatory cytokines, causing several autoimmune-related side effects, especially with chronic use [48,79]. These include drug reaction with eosinophilia and systemic symptoms (DRESS), autoimmune hepatitis, minocycline-induced lupus (MIL) [80] and/or exacerbation of systemic lupus erythematous (SLE), antinuclear antibodies (ANA) and antineutrophil cytoplasmic antibody-associated (ANCA) vasculitis [81], serum sickness-like reaction (SSLR), and other hypersensitivity reactions [21,82]. Cases of hypothyroidism and autoimmune type I diabetes mellitus have also been described as adverse effects due to minocycline [83]. 

DRESS syndrome, also called drug-induced hypersensitivity syndrome (DIHS), is a severe adverse reaction to medications characterized by fever, skin eruption, lymphadenopathy, and facial edema that may occur anytime between 2 weeks and 2 months after initial exposure to the medication. DRESS causes organ inflammation, mainly of the liver, but also the heart, kidneys, lungs, etc., leading to hepatitis, cerebral edema, and/or myocarditis, which results in death in 10% of cases [84,85,86]. Patients with dark skin are more prone to this adverse effect, perhaps because those of African descent have a higher incidence of altered cytochrome P450 metabolism and minocycline is mainly metabolized in the liver [85]. Persistence of symptoms may be due to formation of a minocycline-melanin complex, particularly in those with dark skin containing more melanin [51]. Persistent and severe myocarditis associated with DRESS has also been reported [85,87,88,89,90]. Additional symptoms may present weeks to months after interruption of the causal drug, including myocardial disfunction, cardiomegaly, ECG abnormalities, eosinophilia, and mildly elevated cardiac enzymes [85]. Minocycline or its metabolites may bind myocardial collagen, leading to a T cell-mediated inflammation cascade [85]. Persistent inflammation attracts eosinophils, which damage the cardiac muscle, maybe even causing cardiomyocyte necrosis via increased eosinophil peroxidase [91]. 

Several cases of DRESS-related myocarditis can be found in the research of Morikawa et al. [92]. Loner et al. [89] reported a 21-year-old female patient who had an episode of syncope while in the emergency room and was treated with extracorporeal membrane oxygenation. She spent 20 days in the cardiac ICU and a total of 62 days in the hospital. She was diagnosed with acute necrotizing eosinophilic myocarditis secondary to minocycline-induced DRESS. Wu et al. [90] reported a 46-year-old female patient with a previously unremarkable clinical history who initially presented with rash, fever, and eosinophilia following administration of minocycline to treat perioral dermatitis. The patient died of multiple organ failure six months after her initial symptoms and the autopsy revealed eosinophilic myocarditis. Another fatal case reported by Parneix-Spake et al. [93] described a 15-year-old male patient on minocycline who presented with fever, diffuse pustular eruption, and enlarged lymph nodes. Minocycline was discontinued and the patient was treated with systemic and topical corticosteroids for 2 months. The patient died suddenly two weeks after discontinuing topical corticosteroids and the autopsy showed myocardial necrosis with interstitial eosinophilic infiltrate. Shaughnessy et al. [85] reported a 38-year-old African-American woman who developed a fever and erythematous eruption 3 weeks after starting minocycline for acne treatment. She was hospitalized for 4 weeks and treated with systemic and topical corticosteroids. On the second week of hospitalization she was diagnosed with myocarditis and myocyte necrosis, which was revealed via endomyocardial biopsy. She was treated with prednisone and eventually discharged but required a second hospitalization where a second endomyocardial biopsy revealed an intense infiltrate of lymphocytes and eosinophils with cardiomyocyte necrosis. The authors suggest that given these minocycline-related severe side effects, acne in those of African descent should potentially be preferentially treated with an alternative tetracycline drug. Myocarditis is not the only potential life-threatening pathology associated with DRESS. Lan et al. [94] reported the case of a 13-year-old female patient who developed DRESS 3 weeks after starting minocycline for acne. The treatment consisted of high-dose steroids, but 48 h later the patient developed liver failure and had to be transplanted. 

Viral infections may trigger drug hypersensitivity, leading to DRESS syndrome. For example, Descamps et al. [95] reported an association between human herpesvirus 6 infection and the development of DRESS and suggested that viral infection may interfere with minocycline metabolism via cytochrome P450. Other viruses that have been shown to influence this process are HIV, cytomegalovirus, Epstein–Barr virus and human herpesvirus 7. These viruses may also stimulate proliferation of viral-specific and nonspecific T cells which can cause a massive release of cytokines [88].

Minocycline-induced lupus (MIL) is another serious adverse effect that may be caused by systemic administration of minocycline and other drugs. MIL shows clinical symptoms like those of SLE, being characterized by arthralgia (90% of the cases), fever, arthritis, rash, and, rarely, pneumonitis and cutaneous vasculitis [96]. Patients also test positive for ANA and ANCA [80,97]. To be diagnosed with MIL the patients must test positive for ANA, have no previous clinical history of lupus, and the situation must resolve when the causing drug is discontinued [98]. 

The pathophysiology of drug-induced lupus (such as MIL) depends on the causative drug, but it seems to involve genetic factors [99] and an inflammatory cascade. Drug-induced lupus is usually milder than SLE, with markedly different clinical course and prognosis, although a few life-threatening situations have been reported [96]. Several cases of MIL have been described, with all patients presenting polyarthralgia, polyarthritis, dermatological manifestations such as rash, subcutaneous nodules, alopecia, etc., and ANA-positive tests [100]. Clark et al. [101] reported the case of a male teenager patient who showed an alternative clinical presentation of minocycline-induced lupus with a generalized urticarial eruption that could have been easily mistaken as drug-induced urticaria. Lab results, however, showed positive ANA and the temporal association of minocycline use suggested a case of MIL. Symptoms quickly disappeared when the patient discontinued minocycline and was treated with oral prednisone. 

SSLR appears 6 to 21 days post-administration of the causal drug and is presented by fever, arthralgias, urticaria, and lymphadenopathy. Unlike MIL, patients with SSLR are negative for ANA [101]. The SSLR usually normalizes after discontinuing the drug, but more severe cases may require treatment and hospitalization and can even be life-threatening like DRESS [102,103,104]. Puyana et al. [104] first described this adverse effect in a young male patient who had been taking oral minocycline for 8 days and presented urticaria, fever, lymphadenopathy, and arthralgia. Medication was discontinued and the patient’s symptoms completely resolved 6 days after treatment with antihistamines and steroids. Levenson et al. [103] reported two cases of female patients who had tolerated tetracycline treatments in the past but, upon treatment with minocycline for 2 weeks, developed erythematous dermatitis and arthralgias. Both had to be treated with antihistamines and corticosteroids for the reaction to subside. Malakar et al. [105] reported two other cases of female teenagers being treated with minocycline for acne. Both developed fever, urticaria, and polyarthralgia and both cases resolved after the patients discontinued the treatment and one was treated with a short course of steroids.

Vasculitis, the inflammation of blood vessels, is also a serious, albeit rare, adverse effect of minocycline administration. Vasculitis most commonly presents as polyarteritis nodosa (PAN) [106], a necrotizing vasculitis affecting medium-sized arteries. Systemic symptoms resulting from the involvement of several organs (skin, kidneys, heart, nervous system) include fatigue, fever, loss of appetite and weight, arthralgia, and myalgia [80]. Patients also present with purplish reticular skin rashes mimicking livedo reticularis and/or subcutaneous nodules [106]. Tehrani et al. [107] reported the case of a young female patient who developed minocycline-induced PAN and reviewed all seven cases that had been reported previously, the majority affecting females. The patient had a 2-month history of pink nodules on her legs and elevated ANA titer. She was first diagnosed with SLE, minocycline was discontinued, and the situation evolved favorably over the next 4 weeks without further treatment. Since the pink nodules remained, a biopsy was performed that revealed PAN. All symptoms subsided 8 months after discontinuing minocycline. Gait et al. [79] reported a female patient who presented with tender nodules and brown pigmentation on her limbs with no major systemic symptoms. She had been on minocycline for treatment of acne for 2 years. A skin biopsy led to a diagnosis of PAN with ANCA but not ANA. Minocycline use was discontinued and symptoms resolved after 3 months. 

#### 3.1.5. Anaphylaxis

Anaphylaxis is a type I hypersensitivity reaction reported with minocycline and, although rare, may be life-threatening. This allergic reaction is rapid in onset, with symptoms that include more than one of the following: itchy rash, throat and/or tongue swelling, vomiting, shortness of breath, and low blood pressure, among others [108]. It is mediated by vasoactive amines released from mast cells and basophiles sensitized by IgE [109]. Anaphylactoid symptoms in response to minocycline are extremely rare but at least two cases have been reported. Okano et al. [110] first described these symptoms in a female patient prescribed minocycline for the treatment of salpingitis. Within half an hour of her first dose she developed dyspnea, followed by itching and burning sensations, generalized swelling and erythema, and decreased blood pressure. Jang et al. [109] described a female patient who presented with urticaria, angioedema, dyspnea, and hypotension on three separate occasions. She was treated for anaphylaxis and the causative agent was revealed to be minocycline.

It has been proposed that minocycline-induced anaphylaxis is caused by a derived metabolite, possibly an iminoquinone derivative, which can only be formed from the amino acid side chain of minocycline (not doxycycline nor tetracycline). The derivative can directly bind and damage cell macromolecules or can function as a hapten, leading to an uncontrolled immune response [97], as previously discussed.

#### 3.1.6. Respiratory System-Related 

Minocycline and other drugs have been reported to cause eosinophilic pneumonia (EP), a form of pneumonia where patients present with pulmonary infiltrates and eosinophilia. Bartal et al. [111] reviewed several cases of drug-induced EP described since the syndrome was defined in 1990. Hung [112] reviewed 26 cases of minocycline-induced EP reported prior to 2015. Although the causes and pathogenesis of EP are not known, it seems that eosinophil activation and accumulation in the lungs are mediated by cytokines produced by activated T-lymphocytes [113]. Outcomes of minocycline-induced EP are generally favorable, with symptoms resolving upon treatment discontinuation with or without corticosteroid therapy and no reported lethal cases [112].

Hypersensitivity pneumonitis (HP), an immune-mediated disease, may also be triggered by exposure to minocycline and other drugs, microorganisms, and other antigens [114,115]. HP is characterized by non-immunoglobulin E-mediated inflammation of the lungs, but not all patients develop autoantibodies or have symptoms of autoimmune disease [115]. Symptoms are similar to those of influenza, but diagnosis can be obtained via chest X-ray, which will reveal nodular infiltration, and evaluation of pulmonary function, which will be restrictive. Bronchoalveolar lavage fluid will reveal a marked lymphocytosis [116]. Usually, interrupting treatment with the causative agent resolves symptoms and early diagnosis leads to good prognosis without development of significant restrictive pulmonary function or extensive lung infiltrates. Repeated episodes of HP, however, can lead to nodular pulmonary infiltrates and nonspecific interstitial pneumonia or to idiopathic pulmonary fibrosis [115].

### 3.2. Contraindications

Limited human studies suggest that minocycline may have deleterious effects on spermatogenesis and should not be used by individuals attempting to conceive a child [117]. Minocycline is contraindicated during pregnancy (considered pregnancy category D) because of teratogenicity [8]. Oral minocycline and other tetracyclines cross the placenta and inhibit bone growth, which may lead to skeletal malformations and developmental retardation. Additionally, oral minocycline may cause future teeth discoloration in the infant if taken orally during the second and third trimesters of pregnancy [9,33]. Although “short-term” minocycline use has been deemed safe during lactation, “longer-term” use, as is typical for acne and rosacea treatment, is not recommended for nursing mothers [118]. It is difficult to identify exactly what duration falls under “short-term” and “longer-term” as traditional lactation sources such as LactMed do not define these terms. Topical minocycline used by the mother does not pose a risk to breast-fed infants [118]. Tetracyclines should not be used in children under the age of 9 as they can permanently stain developing teeth, with a prevalence of 3–6% [77]. 

Minocycline should not be administered to patients with hypersensitivity to tetracycline antibiotics since there is a complete cross-sensitivity in this group [33]. Additionally, patients with severe liver function impairment and renal failure should not take minocycline [9,33].

## 4. Interaction of Minocycline with Other Drugs 

Antibiotics are well known to potentially interact with other drugs and lead to side effects when administered systemically [119]. There are 1063 entries for potential drug interactions with minocycline in DrugBank [31]. Drugs.com reports 181 drugs known to interact with minocycline, divided into major (highly clinically significant, avoid combinations), moderate (avoid combinations except in special circumstances), and minor (minimize combined use, consider alternative drugs, institute monitoring plan if taken in combination) interactions [120]. Table 3 summarizes these interactions.

Minocycline may increase or decrease the effects of other medications. For example, minocycline and other tetracyclines may potentiate the hypoprothrombinemic activity of warfarin and other anticoagulants often administered for life. The dosage of the anticoagulant must be adjusted when taking minocycline to avoid hemorrhages. Similarly, minocycline increases the serum levels of digoxin, which is used to treat atrial fibrillation and other heart conditions. The interaction of minocycline with insulin should be carefully considered. Minocycline may enhance the hypoglycemic effect of insulin, a drug that must be administered regularly to diabetic patients, so blood glucose levels must be even more carefully monitored while taking minocycline. The FDA places warnings on some tetracyclines to indicate that their administration may reduce the efficiency of oral contraceptives [121]. However, two studies have shown that there is no statistical difference in unplanned pregnancies or serum levels of estradiol and progesterone between patients concomitantly taking oral contraceptives and tetracyclines vs. oral contraceptives alone [122,123]. It is possible that a small number of women taking systemic tetracyclines may experience reduced serum concentrations of gonadotropins and ovulate even while on oral contraceptives [122]. This is particularly pertinent since minocycline is mainly used to treat women and patients in the age group of 18–44 years old. Although described as a minor interaction, diuretics and minocycline administered concomitantly may cause nephrotoxicity and impair renal function. The concomitant use of minocycline with penicillins should be avoided due to the bacteriostatic effects of minocycline potentially decreasing the bactericidal effects of penicillins.

## 5. Topical Minocycline Development, Safety and Efficacy 

Due to concerns of bacterial resistance and the many side effects associated with systemic minocycline use, development of topical formulations has become a priority. A summary of minocycline topical formulations available in the USA market or undergoing clinical trials is shown in Table 4. Despite the noted advantages of topical delivery of minocycline, this route of administration has been unavailable until the recent FDA approval (October 2019) of the first topical 4% foam (Table 4) [124]. While orally administered minocycline is a solid dosage form, topical minocycline exists in the form of semi-solid formulations, which significantly decreases its stability. Additionally, topically applied minocycline must penetrate the skin stratum corneum and travel against the sebum flow to enter the pilosebaceous unit where *C. acnes* resides and acne begins [82]. Formulation stability and skin permeation are two major challenges that must be overcome when dealing with topical formulations of minocycline. 

### 5.1. Development of Topical Minocycline

There are currently two topical formulations of minocycline available in the market, both topical foams developed by Vyne Therapeutics, 4% minocycline foam (Amzeeq^®^) for the treatment of the inflammatory lesions of acne vulgaris [125,126] and 1.5% minocycline foam (Zilxi^®^) for the treatment of inflammatory lesions of rosacea [127]. Two additional topical minocycline candidates are currently in late clinical investigation with the potential to be important additions to the local treatment paradigm: Hovione’s HY01 for PPR (NCT032632732) and BioPharmX BPX-01 and BPX-04 for acne and PPR, respectively (NCT02815332 and NCT03667222). Hovione’s formulation uses a proprietary emollient excipient composed of anhydrous hydrocarbon-based gelling agents that promote water retention and improve epidermal barrier function [128], an important feature for patients with a compromised skin barrier resulting from PPR. Hovione’s gel stabilizes a novel free-base crystalline form of minocycline, which can potentially reduce skin irritability due to its lower acidity (pH 6–6.5 compared to other products with pH of 3.5–4.5). This gel confers higher stability and higher lipophilicity compared to minocycline hydrochloride [129,130]. BiopharmX uses a proprietary anhydrous hydrophilic gel formulation, HyantX™, which completely solubilizes minocycline hydrochloride and allows rapid absorption into the skin [131]. 

Another mechanism to increase the stability and permeation capability of topical minocycline is the use of advanced delivery systems, novel carriers for minocycline that may stabilize and increase the solubility of the drug, allowing for an easier topical application. These systems remain in the research phase and are mainly related to the treatment of periodontitis [132,133,134]. The development of starch-based nanocapsules with minocycline hydrochloride for topical application has also been described, and the study revealed a long-term physical stability of the formulations [135]. Marto et al. [136] also developed water/oil starch-based Pickering emulsions (emulsion stabilized by solid particles) with minocycline hydrochloride and showed that this system was suitable for topical minocycline hydrochloride administration, allowing for drug solubilization and deposition onto the skin with a prolonged drug release. 

### 5.2. Efficacy of Topical Minocycline

While foam formulations of minocycline are already available in the market, clinical trials are ongoing to test alternate topical formulations developed for the treatment of acne and rosacea. In pooled phase III trials of 4% minocycline foam for treatment of acne, the absolute change in inflammatory lesion count from baseline at week 12 was −13.79 compared to −10.94 for the vehicle (*p* = 0.0001) and the proportion of subjects achieving treatment success according to the Investigator’s Global Assessment (IGA) was 11.51 compared to 6.34 for the vehicle (*p* = 0.0188) [137]. In the phase III trials for minocycline 1.5% foam for treatment of rosacea, the change from baseline to week 12 in inflammatory lesion count was −17.57 compared to −15.65 for the vehicle (*p* = 0.0031) for one study and −18.54 compared to −14.88 for the vehicle (*p* = 0.0001) in the other study. Subjects achieving IGA success were 52.1% vs. 43% of the vehicle (*p* = 0.027) in the first study and 49.1% vs. 39% of the vehicle (*p* = 0.0077) in the second study [138]. 

BiopharmX is evaluating clinical trials of minocycline topical gels BPX-01 [139] and BPX-04 [140] for the treatment of inflammatory acne vulgaris and PPR, respectively [131,141]. To test BPX-01 for treatment of acne, BiopharmX enrolled subjects with moderate-to-severe inflammatory non-nodular acne (n = 226) at 15 centers who were randomized 1:1:1 to treatment with BPX-01 1%, BPX-01 2%, or a vehicle control once daily for 12 weeks. The primary endpoint was reduction in the number of inflammatory lesions; other endpoints included the number of noninflammatory lesions, Investigator’s Global Assessment (IGA) of severity, and subjective ratings of acne. BPX-01 2% reduced the number of inflammatory lesions by 58.5 percent, exceeding the reduction in the vehicle control group (43.8%; *p* = 0.0256) [141].

A randomized, double-blind, vehicle-controlled phase IIb trial for BPX-04 to treat PPR enrolled 206 subjects aged 18 years and above with moderate-to-severe PPR across 11 sites in the United States. The study evaluated the safety and efficacy of once daily application of BPX-04, a 1% minocycline gel, versus a vehicle control over a 12-week treatment period. The study was designed to demonstrate a statistically significant mean change in the number of facial inflammatory lesions from baseline to week 12 and in the proportion of subjects with a two-grade improvement to clear or almost clear on the IGA scale from baseline to week 12. The mean change in the number of lesions from baseline to week 12 was −13.6 with BPX-04 and −10.3 with the vehicle alone (*p* = 0.004). The proportion of subjects with a two-grade improvement in IGA to 0 (clear) or 1 (almost clear) from baseline to week 12 was 52.3% with BPX-04 compared to 32.3% with the vehicle only (*p* = 0.018) [131]. In June 2019, BioPharmX announced that BPX-04 had met primary and secondary endpoints with statistical significance [131].

Hovione also completed phase IIb studies for minocycline 1% and 3% gel (MARS—Minocycline Against Rosacea Study) and selected 3% minocycline gel for phase III studies. A total of 270 patients with PPR across 26 sites in the United States were enrolled in the MARS [130]. The 12-week, randomized, double-masked, parallel group, vehicle-controlled study tested 1% minocycline and 3% minocycline efficacy outcomes in mean change in inflammatory lesions at week 12 from baseline and proportion of patients achieving “clear” or “almost clear” together with a two-grade reduction in the IGA. HY01 statistically reduced the number of inflammatory lesions at week 12 compared to the vehicle in a dose-dependent manner. Lesion counts reduced 12.6, 13.1, and 7.9, respectively, in 1% (*p* = 0.01), 3% (*p* = 0.007), and vehicle groups. The proportion of patients achieving IGA success was 39% in the minocycline 1% arm (*p* = 0.335), 46% in the 3% arm (*p* = 0.038), and 31% in the vehicle arm. MARS recorded a low number of treatment-related adverse events with only 3% and 5% of patients reporting an event in the 1% and 3% groups, respectively [130]. 

If approved, these equally promising minocycline topical formulations to treat the inflammatory lesions of rosacea from BiopharmX and Hovione may provide a targeted alternative to systemic doxycycline, minocycline, and other antibiotics [142,143]. 

Topical administration of minocycline has the advantage of providing much higher drug concentration and persistence in skin layers when compared to oral administration [144]. Topical minocycline requires a much lower amount of drug to achieve similar integumentary levels compared with the amount required via oral administration [145]. Further, minocycline is effective in eradicating topical pathogens that cause superficial infections, especially those caused by Gram-positive bacteria [146,147]. Importantly, this includes multiresistant strains such as methicillin-resistant *Staphylococcus aureus,* the leading cause of skin and soft tissue infections [146]. Topically applied minocycline also carries the advantage of avoiding the many possible side effects and interactions associated with the systemic use of the antibiotic.

### 5.3. Pharmacokinetics of Topical Minocycline

The pharmacokinetics of topical minocycline differ from systemic minocycline [130,148]. Jones et al. [148] reported a study with 30 adults with moderate-to-severe acne comparing the systemic effects of single-dose oral administration (1 mg/kg) of extended-release Solodyn^®^ capsules, with a multidose (total of 4 g) topical administration of 4% minocycline hydrochloride. Systemic exposure resulting from daily topical application of the foam for 21 days was 730–765 times lower than from single-dose oral administration. Hovione performed a pharmacokinetic evaluation in the phase 2B MARS study comparing minocycline systemic levels at baseline and at week 12 in a subset of the study population consisting of 30 patients. The lower limit of quantification (LLOQ) was 0.999 ng/mL. Plasma concentrations were detected in few patients in each group and in those the mean AUC was 0.023 h·µg/mL in the 1% arm and 0.021 h·µg/mL in the 3% arm. Systemic levels of topical minocycline at week 12 were more than 1000-fold lower than those of extended-release Solodyn^®^ capsules, clearly showing the pharmacokinetic advantages of topical delivery [130].

### 5.4. Safety of Locally Administered Minocycline Using Topical Formulations

While systemic treatment with minocycline can result in interactions with concomitant drugs, there are only six drug interactions with topical minocycline currently reported on Drugs.com [9]. Topical formulations of minocycline have the advantage of a targeted local application, resulting in enhanced skin bioavailability and efficacy, while simultaneously avoiding the adverse effects observed with oral and IV formulations [137]. Adverse effects caused by topical formulations of minocycline have been described but are not as severe as those caused by systemic minocycline. Patients on 4% minocycline foam experienced mild-to-moderate erythema, dryness, hyperpigmentation, skin peeling, and itching, but rates of these effects were similar to the vehicle alone [149]. For patients in phase III trials for 1.5% minocycline foam for treatment of rosacea, most adverse events were mild to moderate, with the most common noncutaneous adverse event being viral upper respiratory tract infection and the most common cutaneous adverse event being pruritus. Only one patient discontinued because of pruritus, which resolved after discontinuation. Skin tolerability issues at the application site were reported as none or mild for more than 80% of the participants [138]. BPX-01 treatment was well-tolerated, with no photosensitivity, hyperpigmentation, or skin discoloration reported [139]. Some adverse events of BPX-04 included upper respiratory tract infection (5.3%), gastroenteritis (2.4%), and headache (2.4%), with the majority determined to be not treatment-related [131]. In the phase IIb trial for HY-01 there were single reports of application site dermatitis and pruritis with the 1% formulation and single reports of nausea, application site erythema and pruritis, hypersensitivity, headache, rosacea, and urticaria with the 3% formulation [130]. To our knowledge, only one study reported a serious adverse effect, eosinophilic pneumonia, caused by topical minocycline for treatment of acne [150].

### 5.5. Bacterial Resistance to Topical Minocycline

Practicing good antibiotic stewardship means being attuned to the possibility of inducing bacterial antibiotic resistance and, when an oral antibiotic is necessary, choosing a narrow-spectrum oral antibiotic. At times, when an oral antibiotic is not indicated and the practitioner is choosing a topical antibiotic, one must again consider the implications of inducing bacterial resistance and altering the skin microbiome. As mentioned above, use of topical clindamycin, erythromycin, azithromycin, or clarithromycin, once commonplace in the acne treatment paradigm, has been jeopardized by increasing *C. acnes* resistance to these drugs [23,151]. In contrast, *C. acnes* strains displayed a low propensity for developing resistance to minocycline [152,153], with frequencies of resistance after a single exposure of less than 10^−8^ (or 1 in 100 million) at 2 to 16 times the minimum inhibitory concentration [153]. Additionally, when given topically, minocycline reaches concentrations that are well outside of the mutant selection window, thus further minimizing the likelihood of bacterial resistance developing [152]. These findings allowed for updated labeling on minocycline 4% foam that states that *C. acnes* strains display a low propensity for development of resistance to minocycline [154]. 

## 6. Conclusions

Tetracyclines, especially minocycline and doxycycline, have historically been the most commonly prescribed oral antibiotics to treat dermatological conditions such as acne and rosacea. However, there are several adverse effects associated with oral administration of these second-generation tetracyclines, which, especially in the case of minocycline, may be severe. Although rare, some of the side effects may be fatal. These serious effects led the Drug Therapy Bulletin to publish an editorial entitled “Time to say goodbye to minocycline?” [155] which states that there is no clinical advantage that justifies the prescription of oral minocycline for treating acne [43]. The National Institutes for Health and Care Excellence recommended that physicians avoid prescribing oral minocycline [156]. Although the newest third-generation tetracycline, sarecycline, offers a narrow spectrum approach to treating acne, some patients require a topical rather than an oral antibiotic. To add to this, recently approved or under-development topical formulations of minocycline constitute a promising safer alternative to oral minocycline for treating acne and rosacea while avoiding serious side effects of systemic minocycline. Topical delivery of minocycline should be considered when treating dermatological diseases such as acne and rosacea [157]. In summary, this paper reviewed the adverse effects caused by oral administration of minocycline, focusing on the most severe, and highlighted the improved risk–benefit ratio of locally administered topical formulations for treatment of acne and rosacea.

## Figures and Tables

**Table 1:** Brands of minocycline currently approved for systemic use in the USA market [8,9,32].

Brand	Dosage Forms	Manufacturer	Indications
Cleeravue-M	Oral tablets, extended release	StoneBridge Pharma	Moderate-to-severe acne vulgaris.
Dynacin	Oral capsule	Medicis, The Dermatology Co.; Par Pharmaceutical, Inc.	Acne, Rocky Mountain spotted fever, typhus fever, Q fever, tick fevers, respiratory tract infections, lymphogranuloma venereum psittacosis, trachoma, inclusion conjunctivitis, nongonococcal urethritis, endocervical, or rectal infections, relapsing fever, chancroid, plague, tularemia, cholera, brucellosis, bartonellosis, granuloma inguinale, etc.
Minocin	Oral capsule (pellet-filled) IV solution (reconstituted)	Bausch Rempex Pharms	Same as Dynacin.
Minolira	Oral tablet, extended release (24 h)	EPI Health	Inflammatory lesions of non-nodular moderate-to-severe acne vulgaris in patients 12 years of age and older.
Solodyn	Oral tablet, extended release (24 h)	Medicis	Inflammatory lesions of non-nodular moderate-to-severe acne vulgaris in patients 12 years of age and older.
Ximino	Oral capsule, extended release (24 h)	Journey Medical Corporation	Inflammatory lesions of non-nodular moderate-to-severe acne vulgaris in patients 12 years of age and older.
Arestin	Oral Powder (microspheres)	OraPharma	Periodontitis
Generic	Oral tablet Oral capsule Oral tablet, extended release (24 h)	Several labs	Same as the brand minocycline formulations.

**Table 2:** Possible adverse effects caused by the systemic use of minocycline [9,49,53].

Target	Very Common (>10%)	Common (1–10%)	Rare (0.01–1%)	Very Rare (<0.01%)	Others and/or Frequency Not Reported
Nervous system	Headache (up to 23%)	Dizziness, somnolence, tinnitus, vertigo, mood alteration	Hypoesthesia, IIH, paresthesia, intracranial hypertension, impaired/decreased hearing, sedation, ataxia, vestibular reactions	Bulging fontanels (in infants)	Convulsions
Skin		Urticaria, rash, pruritus, erythematous rash	Angioedema, alopecia, erythema, fixed drug eruptions, hyperpigmentation, photosensitivity, cutaneous vasculitis, maculopapular rash, DRESS	Exfoliative dermatitis, hyperpigmentation of nails/nail beds, Stevens–Johnson syndrome, toxic epidermal necrolysis	Sweet’s syndrome (acute febrile neutrophilic dermatosis), anaphylactoid purpura, angioneurotic edema
Gastrointestinal system		Nausea, vomits, teeth discoloration, diarrhea, abdominal pain	Dry mouth, dysphagia, dyspepsia, colitis,	Candidiasis, enamel hypoplasia, enterocolitis, esophagitis, esophageal ulcerations, glossitis, pancreatitis, oral mucosa discoloration	Inflammatory lesions in the oral and anogenital regions
Musculo- skeletal system		Arthralgia, myalgia, MIL, arthritis	Joint stiffness, joint swelling, myopathy, hypersensitivity-associated rhabdomyolysis	Joint discoloration	Severe acute myopathy
Hepatobiliary system		Abnormal hepatic function, hepatitis	Increased liver enzymes, autoimmune hepatitis, hepatic cholestasis, hepatic failure, hyperbilirubinemia, jaundice, liver injury	Fulminant hepatitis	Autoimmune hepatitis with lupus-like symptoms, acute hypersensitivity hepatitis associated with eosinophilia and dermatitis
Respiratory system		Dyspnea	Cough, interstitial lung disease, pulmonary infiltration, eosinophilic pneumonia, bronchospasm, HP, pneumonitis, pleural effusions	Exacerbation of asthma	Pulmonary lupus, relapsing acute respiratory failure
Immune system		Hypersensitivity	SSLR, ANCA-positive vasculitis	Immunosuppression	Positive ANCA titers, polyarteritis nodosa, ANCA-positive crescentic glomerulonephritis, autoimmune hepatitis, necrotizing vasculitis and systemic reactions
Blood and lymphatic systems			Eosinophilia, leukopenia, neutropenia, thrombocytopenia, hemolytic anemia, pancytopenia, agranulocytosis	Thrombocytosis	ANCA-positive vasculitis,
Cardiovascular system			Palpitations, tachycardia, myocarditis, pericarditis, cardiac arrest, polyarteritis nodosa, vasculitis, hypotension, hypertension	Acute cardiac failure	
Metabolic system		Anorexia	Dehydration	Hyperphosphatemia	Acidosis in patients with renal dysfunction
Endocrine system			Hyperthyroidism, thyroiditis, hypothyroidism, autoimmune thyroiditis	Brown-black microscopic thyroid discoloration	Discolored breast secretions
Renal and genitourinary systems			Acute kidney injury, azotemia, increased blood and serum urea, interstitial nephritis, acute renal failure	Balanitis, vulvovaginitis	Deleterious effects on spermatogenesis
Others (hypersensitivity reactions)			Anaphylaxis/anaphylactoid reaction (including shock, death)		Pulmonary infiltrates, night sweats, fever, eosinophilia, severe CNS -pulmonary HSR, EP with relapsing acute respiratory failure, late-onset drug fever

ANA, antinuclear antibody; ANCA, antineutrophil cytoplasmic antibody; CNS, central nervous system; EP, eosinophilic pneumonia; MIL, minocycline-induced lupus; DRESS, drug rash with eosinophilia and systemic symptoms; HP, hypersensitivity pneumonitis; HSR, hypersensitivity syndrome reaction; IIH, idiopathic intracranial hypertension; SLE, systemic lupus erythematosus; SSLR, serum sickness-like reaction.

**Table 3:** Interactions of minocycline with other drugs [120].

Drug	Drug Type	Effect
**Major drug interactions—highly clinically significant, avoid combinations**		
Acitretin, etretinate, isotretinoin, tretinoin, vitamin A	Retinoids, vitamin A derivatives	IIH caused by increased pressure in the brain, may lead to permanent vision loss
Aminolevulinic acid	Endogenous non-proteinogenic amino acid	Both drugs increase photosensitivity and may lead to severe sunburns
BCG	Bacillus Calmette–Guérin (BCG vaccine)	Minocycline reduces the antitumor activity of BCG in the bladder
Cholera vaccine (live) Typhoid vaccine (live)	Vaccines	Minocycline may reduce the effect of the vaccine
Leflunomide	Immunosuppressive disease-modifying antirheumatic drug	Liver injury
Lomitapide	Enzyme inhibitor	
Mipomersen	Antisense oligonucleotide inhibitor of apo B (cholesterol-lowering)	
Pexidartinib	Tyrosine kinase inhibitor	
Teriflunomide	Active metabolite of leflunomide	
Methoxyflurane	Ether, anesthetic	Kidney problems
**Moderate drug interactions—avoid combinations except in special circumstances**		
Aluminum, calcium, magnesium salts		Interfere with minocycline absorption
Aminophylline	Compound of the bronchodilator theophylline with ethylenediamine (ratio 2:1)	Minocycline increases the effect of aminophylline
Penicillin and derivatives	Penicillins	Minocycline may decrease the effect of penicillins
Anisindione	Synthetic anticoagulant	Minocycline may increase the hypoprothrombinemic effects of warfarin and similar anticoagulants.
Dicoumarol	Natural anticoagulant	
Warfarin	Derivative from dicoumarol	
Asparaginase	Enzyme (treatment of leukemia)	Liver injury
Bedaquiline	Enzyme inhibitor (treatment of multidrug-resistant TB)	
Brentuximab vedotin	Antibody-drug conjugate (treatment of some lymphomas)	
Clofarabine	Purine nucleoside antimetabolite (cancer treatment)	
Daclizumab	Humanized monoclonal antibody (treatment of relapsing forms of MS)	
Efavirenz	Antiretroviral (AIDS treatment)	
Epirubicin	Anthracycline (cancer treatment)	
Idelalisib	Enzyme inhibitor (blood cancer treatment)	
Interferon-β	Cytokine (MS treatment)	
Naltrexone	Opiate antagonist (treatment of addictions)	
Remdesivir	Nucleotide analogue prodrug, antiviral	
Thioguanine	Anticancer chemotherapy drug	
Trabectedin	Chemotherapy drug	
Atracurium, cisatracurium, mivacurium, pancuronium, rocuronium, succinylcholine, vecuronium	Neuromuscular blocking agents	Minocycline may increase the effect of these drugs, leading to respiratory depression and muscle weakness
Balsalazide	Anti-inflammatory	Minocycline may reduce the effect of balsalazide
Bismuth-, iron- or zinc-containing preparations, lanthanum salts		Chelation of minocycline, which may reduce its effective concentration
Digitoxin	Natural cardiac glucoside (cancer therapy)	Minocycline may increase the serum levels of digitoxin and digoxin
Digoxin	Natural cardiac glucoside (treatment of heart conditions)	
Dihydroergotamine, ergonovine, ergotamine, methylergonovine, methysergide maleate	Ergot alkaloids (vasoconstrictors)	Tetracyclines may increase the plasma concentrations and toxicity of ergot alkaloids, leading to liver injury
Ethinyl estradiol	Estrogen (birth control)	Minocycline and other antibiotics may impair the contraceptive effect of estrogens in some rare individuals
Insulin and analogues	Hormone	Minocycline may enhance the hypoglycemic effect of insulin
Methotrexate	Chemotherapy agent, immunosuppressant	Tetracycline may elevate or reduce serum methotrexate concentrations
Methoxsalen	Psoralen (photosensitizing agent)	Increased photosensitivity
Methyl aminolevulinate (topical)	Prodrug (photosensitizing agent)	
Porfimer	Mixture of porphyrin oligomers (photosensitizing agent)	
Verteporfin	Benzoporphyrin derivative (photosensitizing agent)	
Mycophenolate mofetil	Immunosuppressant	Minocycline may reduce the immunosuppressive effects of mycophenolic acid
Oxtriphylline	Salt of choline and theophylline (bronchodilator)	Minocycline may decrease theophylline plasma clearance and increase theophylline levels
Theophylline	Bronchodilator	
Sodium acetate, bicarbonate, citrate, lactate Thrometamine	Organic amine proton acceptor (treatment of metabolic acidosis)	These compounds may decrease the effect of minocycline due to alkalinization of the urine
**Minor drug interactions—minimize combined use, consider alternative drugs, if combined, institute monitoring plan**		
Acetazolamide, amiloride, bendroflumethiazide, benzthiazide, bumetanide, chlorothiazide, chlorthalidone, dichlorphenamide, ethacrynic acid, furosemide, glycerine, hydrochlorothiazide, indapamide, mannitol, methazolamide, methyclothiazide, metolazone, polythiazide, spironolactone, torsemide, triamterene, trichlormethiazide	Diuretics	Decreased renal function
Colestipol	Bile acid sequestrant (lowers blood cholesterol)	May reduce absorption of minocycline
Didanosine	Antiretroviral	
Lithium	Treatment of bipolar and depressive disorders	Minocycline may increase the plasma concentrations of lithium. Rarely, IIH has been reported when minocycline is co-administered with lithium

BCG, bacillus Calmette–Guérin; IIH, idiopathic intracranial hypertension; MS, multiple sclerosis.

**Table 4:** Description of topical minocycline formulations commercially available or under clinical development.

Brand/Phase	Manufacturer	Dosage forms	Minocycline Form	Indications
Amzeeq (FMX101)/market	Vyne Therapeutics	Topical foam (4%)	Minocycline HCl	Treatment of inflammatory lesions of non-nodular moderate-to-severe acne vulgaris in patients 9 years of age and older.
Zilxi/market	Vyne Therapeutics	Topical foam (1.5%)	Minocycline HCl	Treatment of inflammatory lesions of rosacea in adults.
BPX-01/IIb	BiopharmX	Hydrophilic gel (1% and 2%)	Minocycline HCl	Treatment of acne vulgaris
BPX-04/IIb	BiopharmX	Hydrophilic gel (1% and 2%)	Minocycline HCl	Treatment of papulopustular rosacea
HY01/IIb	Hovione	Anhydrous gel (1% and 3%)	Crystalline minocycline base	Treatment of papulopustular rosacea

## Data Availability

Not applicable.

## Abbreviations

ANCA: antineutrophil cytoplasmic antibody-associated; *C. acnes*, *Cutibacterium acnes*; CNS, central nervous system; DIHS, drug-induced hypersensitivity syndrome; DRESS, drug reaction with eosinophilia and systemic symptoms; EP, eosinophilic pneumonia; FDA, Food and Drug Administration; HP, hypersensitivity pneumonitis; HSR, hypersensitivity syndrome reaction; IIH, idiopathic intracranial hypertension; MIL, minocycline-induced lupus; MH, minocycline hydrochloride; PAN, polyarteritis nodosa; PPR, papulopustular rosacea; PTC, pseudotumor cerebri: SLE, systemic lupus erythematosus; SSLR, serum sickness-like reaction, TDS, transient depersonalization syndrome.
